# Single and combined effect of kinesio tape and warm-up on sprint cycling performance

**DOI:** 10.1186/s13102-021-00310-3

**Published:** 2021-07-26

**Authors:** Alessio Rossi, Damiano Formenti, Luca Cavaggioni, Giampietro Alberti, Fabio Esposito, Fabio D’Angelo, Athos Trecroci

**Affiliations:** 1 Department of Computer Science, University of Pisa, Pisa, Italy; 2 Department of Biotechnology and Life Sciences (DBSV), University of Insubria, Varese, Italy; 3 Department of Biomedical Sciences for Health, Università degli Studi di Milano, Milan, Italy; 4 Department of Endocrine and Metabolic Diseases, Obesity Unit and Laboratory of Nutrition and Obesity Research, IRCCS Istituto Auxologico Italiano, Milan, Italy

**Keywords:** Pedaling, Warm-up, Power, Anaerobic, Performance

## Abstract

**Background:**

The fact that kinesio tape may be capable to enhance muscle power would qualify it as practical tool to be considered during passive warm-up (WU) or coupled with active WU processes prior to power-based performance. Therefore, the aim of this study was to investigate the single and combined effect of kinesio tape (KT) and WU on sprint cycling performance.

**Methods:**

In a repeated measure design, fifteen participants underwent six sessions to assess sprint cycling performance involving a combination of three taping conditions (without KT: NoKT; with KT positioned vertically over the thigh muscles KT; with KT positioned horizontally over the thigh muscles: Sham) with two pre-exercise routines (with WU: WU; without WU: NoWU) in a randomized order. Allometric scaling of peak power (PP) and average power (AP) values were considered for each sprint.

**Results:**

KT-WU demonstrated the highest PP and AP with respect to the other conditions (*p* < 0.05), except for AP that was similar to Sham-WU (*p* > 0.05). Moreover, NoKT-NoWU showed the lowest PP and AP with respect to the other conditions (*p* < 0.05).

**Conclusions:**

Overall, our findings suggest that kinesio tape might be a possible tool to be combined with an active WU routine, inducing benefit on sprint performance. Moreover, KT may be considered a potential strategy to include within a passive WU, perhaps where an active WU is not feasible. However, as the influence of KT on muscle function is still unclear, our results should not be overinterpreted.

## Introduction

Warm-up (WU) represents one of the most employed pre-exercise routines anticipating a physical activity, both in health-related and competitive sporting environments [1, 2]. It is widely considered effective to promote specific effects related (e.g., increased thermoregulatory strain and decreased resistance of muscles and joints) and non-related (e.g., post activation potentiation and psychological effects) to temperature changes [1]. Typically, warm-up may occur actively and passively with the former and latter being capable to enhance muscular function [2], eliciting potential improvements in performance. An active warm-up is exercise-mediated with an individual being subjected to a broad spectrum of loads (e.g., physiological, metabolic, neuromuscular, cardiovascular, mechanical, and cognitive) [1, 2] in the attempt to increase her or his readiness for an immediate performance. Whereas, passive recovery aims to promote skin, muscle, core, and body temperature without imposing any load on the same individual.

Specifically, passive modality exploits external sources to apply heat on the body tissues. For instance, Avelar et al. [3] compared the effects of passive (using a thermal blanket applied on participants’ thighs and legs) and active warm-up (induced by whole-body vibration added to squat exercises) modalities on sprint cycle exercise. The authors found no significant differences in the sprint performance variables (e.g., mean and peak power) between the conditions [3], indicating that passive and active WU methods were both able to improve performance. Similarly, Gogte et al. [4] observed the same effectiveness on vertical jump and dynamic balance between active (cycling, leg press, and jump exercises), passive (moist heating applied on lower limbs), and combined warm-up strategies.

Kinesio taping (KT) technique has been extensively studied in recent years due to its potential therapeutic effects not only in rehabilitation and sport medicine, but also in sport performance environment. Among its effects, KT is attributed to enhance muscle power and strength of the treated muscle bellies [5], to relieve pain [6], and to promote skin blood and lymphatic microcirculation [7]. In particular, the supposed capability to influence microcirculation may potentially mediate changes in skin temperature distribution, which is associated to subcutaneous perfusion [8]. Likely skin temperature increases might contribute to minimize the gradient between skin and muscle temperature in the attempt to limiting muscle temperature drop [9]. In this wake, Slomka et al. [8] observed the potential role of KT to influence skin temperature at the site of application. Based on a thermographic analysis, the authors revealed a significant skin temperature decrease after the KT removal while no changes were observed in the placebo group.

The fact that KT might potentially mediate skin temperature changes together with its supposed capability to enhance muscle power and strength would qualify it as possible tool to be considered during passive WUs. Additionally, its higher practicality, compared with other methods (e.g., moist heat application and hot water baths) may also suggest its use coupled with an active WU to influence power-based performance. Although coupling active and passive WU has previously failed to observe additional positive effects on sprint cycling performance improvements [10], the literature is still scant on this topic. Indeed, whether a combination of active and passive WUs would furtherly impact a subsequent maximal performance remains unclear. Furthermore, no studies have even investigated the role of KT in the pre-exercise routine (i.e., WU). On the other hand, it has been observed its usefulness to acutely improve sprint cycling performance and to mitigate performance decrements between 6-s maximal sprints cycling interspersed by 30 min of rest (Trecroci et al. [11]). These results contribute to place emphasis on the potential role of KT within the WU process.

In the light of these considerations, the aim of the present study was to investigate the single and combined effect of KT application and WU on sprint cycling performance. To study potential single and combined effect on sprint cycling performance, we designed an experiment comparing the combination of different KT conditions (with and without KT) and pre-exercise routine (with and without WU). We hypothesized that the use of KT would promote performance benefit on maximal sprint cycling irrespective of pre-exercise routine.

## Materials and methods

### Subjects

Fifteen physically active males participated voluntarily in the study (age: 23.0 ± 1.5 years; body mass: 70.1 ± 9.0 kg; height: 1.81 ± 0.22 m). All participants have no known presence of illness or disease and musculoskeletal injury in the lower limbs within a year prior to the study. Additionally, the participants followed a weekly training routine of 6 h for at least two months. Participants were thoroughly informed of the protocols and procedures before their participation, and written informed consent was provided from them. According to the Helsinki declaration, the Ethical Committee of the local university approved the study.

### Kinesio Tape application

Standard 5 cm black Kinesio tape (KT Tex Gold) was used in both KT and Sham conditions. In KT condition, the tape was applied following the I-shaped KT technique [12] with vastus lateralis and vastus medialis being longitudinally taped with 50 % tension [11, 13, 14]. In Sham condition, two I-shaped strips were applied horizontally with no tension on the frontal surface of the thigh muscles. The KT length was dependent on the femur length of the participants. The overall procedure for KT and Sham conditions mirrored that previously adopted in literature [11, 13, 15, 16]. All applications were administered by the same experienced operator at the beginning of each testing session, immediately before warming up.

### Experimental procedures

**Fig. 1:**
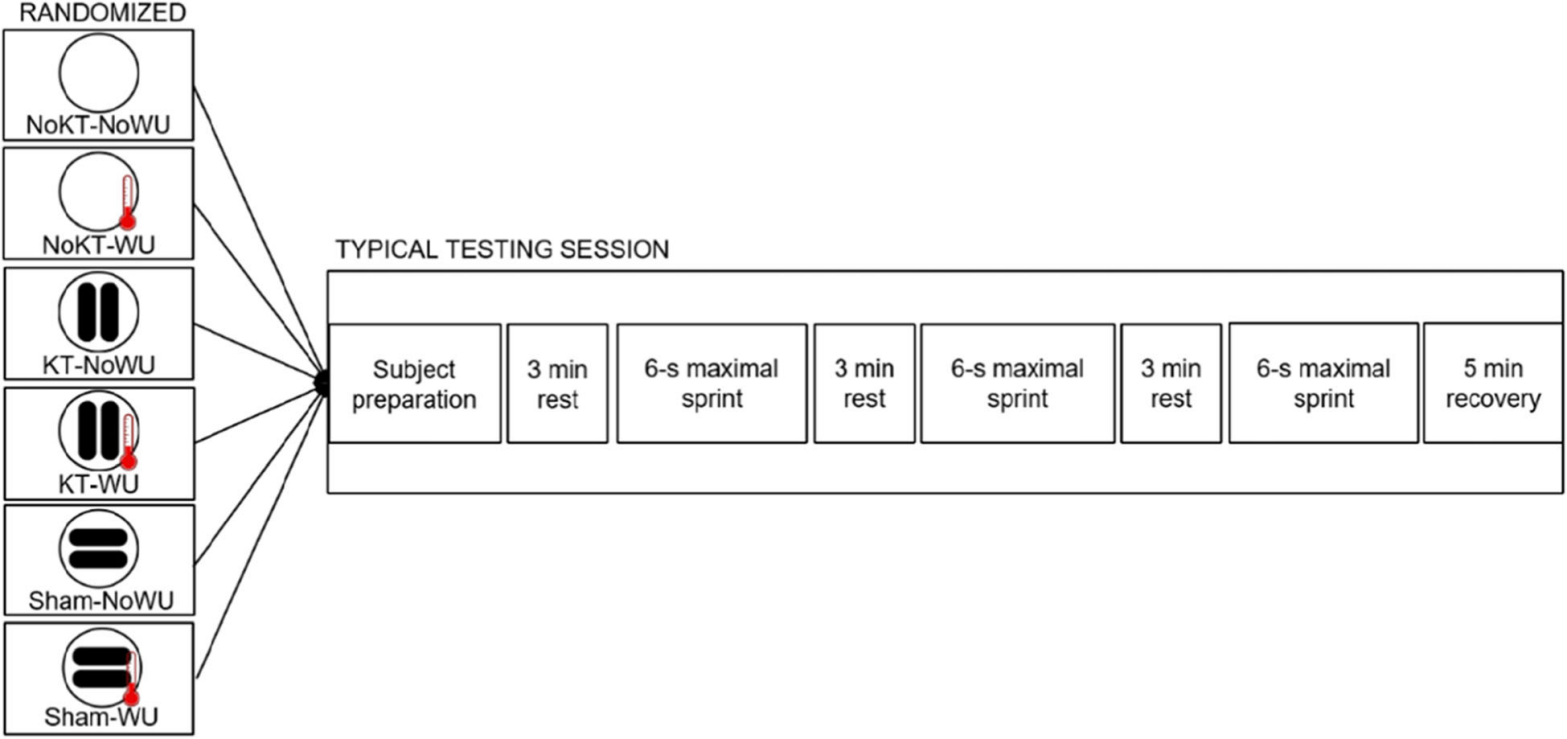
Schematic representation of the experimental design. Note: KT = kinesio taping; WU = warm-up

Two preliminary sessions separated by 5 days were scheduled at the same time of day (from 10 AM to 11.35 AM) to get the participants accustomed with all experimental procedures [17]. In the first session, height and body mass were recorded. During each session, participants trialed each sprint on the same cycle ergometer (Monark 894E, Monark Exercise AB, Vansbro, Sweden), which were customized for each of them by saddle height [18] and initial frictional force. Specifically, the cycle ergometer was equipped with a friction-braked system consisting of a cord sliding against a flywheel. The cord was connected to a built-in weight basket system equivalent, which was set to 10 % of an individual’s body mass. At resting position, the weight basket was locked in its upper position. Concurrently with the sprint, the weight basket was automatically release as soon as the participants reached 100 rpm. This was run by a dedicated software (Monark ATS software v. 3.3, Monark Exercise AB, Vansbro, Sweden), which also recorded the all-performance data during each sprint. After the two preliminary sessions, all participants underwent six experimental sessions to assess sprint cycling performance involving a combination of three taping conditions (NoKT, KT, and Sham) with two pre-exercise routines (WU and NoWU). Experimental design is shown in Fig. 1. To deal with potential order and carryover effects, the experimental sessions were counterbalanced and separated by 5 days. The calculation of the test-retest reliability by the intraclass correlation coefficient (ICC) reported high value of reliability for PP (ICC = 0.95) and AP (ICC = 0.97) between the two sessions All the experimental sessions were performed in a laboratory with a temperature of 22–23 °C, relative humidity of 50 ± 5 %, with no direct ventilation. Conditions and sessions (lasting ~ 60 min each) were separated by 5 days to limit carryover effects due to fatigue-related decay and possible residual effects of the tape [11, 13]. All participants were instructed not consuming caffeinated drinks and not exercising within 24–48 h before testing, respectively.

### Experimental protocol

In the WU sessions, participants warmed up actively via a specific standardized cycling task. Specifically, the WU consisted of 4 min of cycling at ~ 100 W. Near the end of the second, third and fourth min, participants performed a 2-s maximal sprint. At the end of the last sprint bout (fourth min), they remained seated on the cycle ergometer for further 4 min of recovery. In the no-WU sessions, participants did not warm up prior sprinting. The sprinting protocol consisted of 3 × 6-s maximal sprints interspersed by 3 min of rest. Specifically, each participant started pedaling with a moderate cadence (i.e., 60 ± 10 rpm). After 10 s, they were required to increase the cadence of 20 rpm until a 5-s countdown to sprint maximally. During sprinting, all participants were verbally encouraged. After each 6-s sprint, participants remained seated on the cycle ergometer. Peak power (PP) and average power (AP) were recorded during all sprints. The highest values of the three sprints in each condition were considered in the analysis. The 95 % of this value was considered as the criterion score of all subsequent sprints [17]. The achievement of at least 95 % of the criterion score was required in each individual sprint to prevent possible pacing strategies thus guaranteeing a good reliability among the three sprints. If the criterion score was not attained, participants were asked to rest for a further 5 min and restart the session [17]. However, this circumstance did not occur.

### Statistical analysis

All the descriptive statistics are reported as mean ± standard deviation (SD). The PP and AP were scaled using allometric exponents by the log-linear regression approach. The equation y = a × body mass^-b^ (y: PP and AP normalized; a: PP or AP not normalized; b: constant) [19]. The normality of data distribution was assessed by the Shapiro-Wilk’s Normality Test. A one-way analysis of variance for repeated measures was used to detect differences between conditions. Moreover, Bonferroni post-hoc test was performed in order to make pairwise comparison among conditions. Magnitude of the difference was assessed by Cohen’s *d*-value effect size (ES) [20]. The corresponding ES thresholds for *trivial*, *small*, *moderate*, and *large* effects were classified as < 0.2, 0.2 < ES < 0.5, 0.5 < ES < 0.8, and > 0.8, respectively. The statistical significance was set as 0.05. All the analysis was conducted by using Python 3.8 programming language.

## Results

**Fig. 2:**
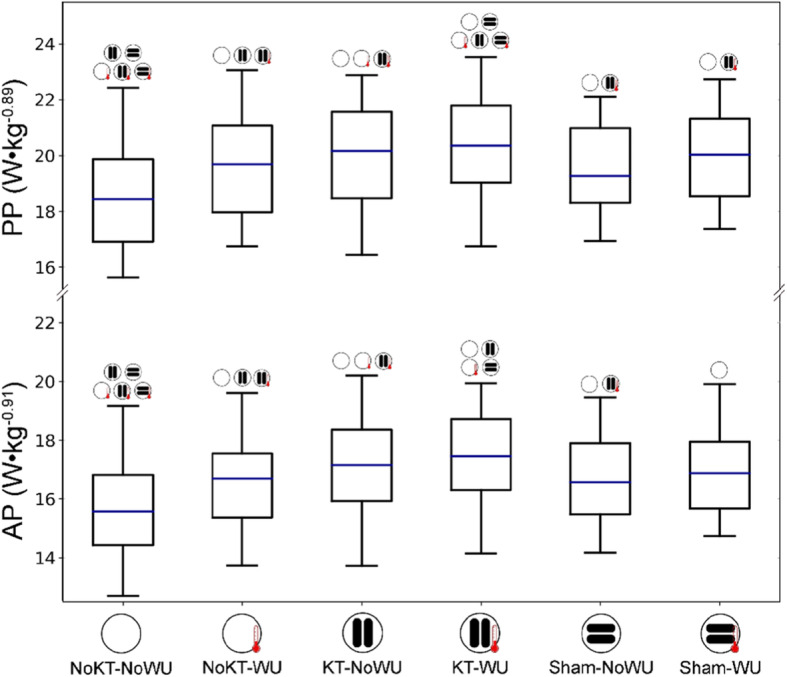
Boxplots describing each condition. The symbols above each boxplot refer to the significant difference with respect to the specific condition. Note: PP = peak power; AP = average power

**Fig. 3:**
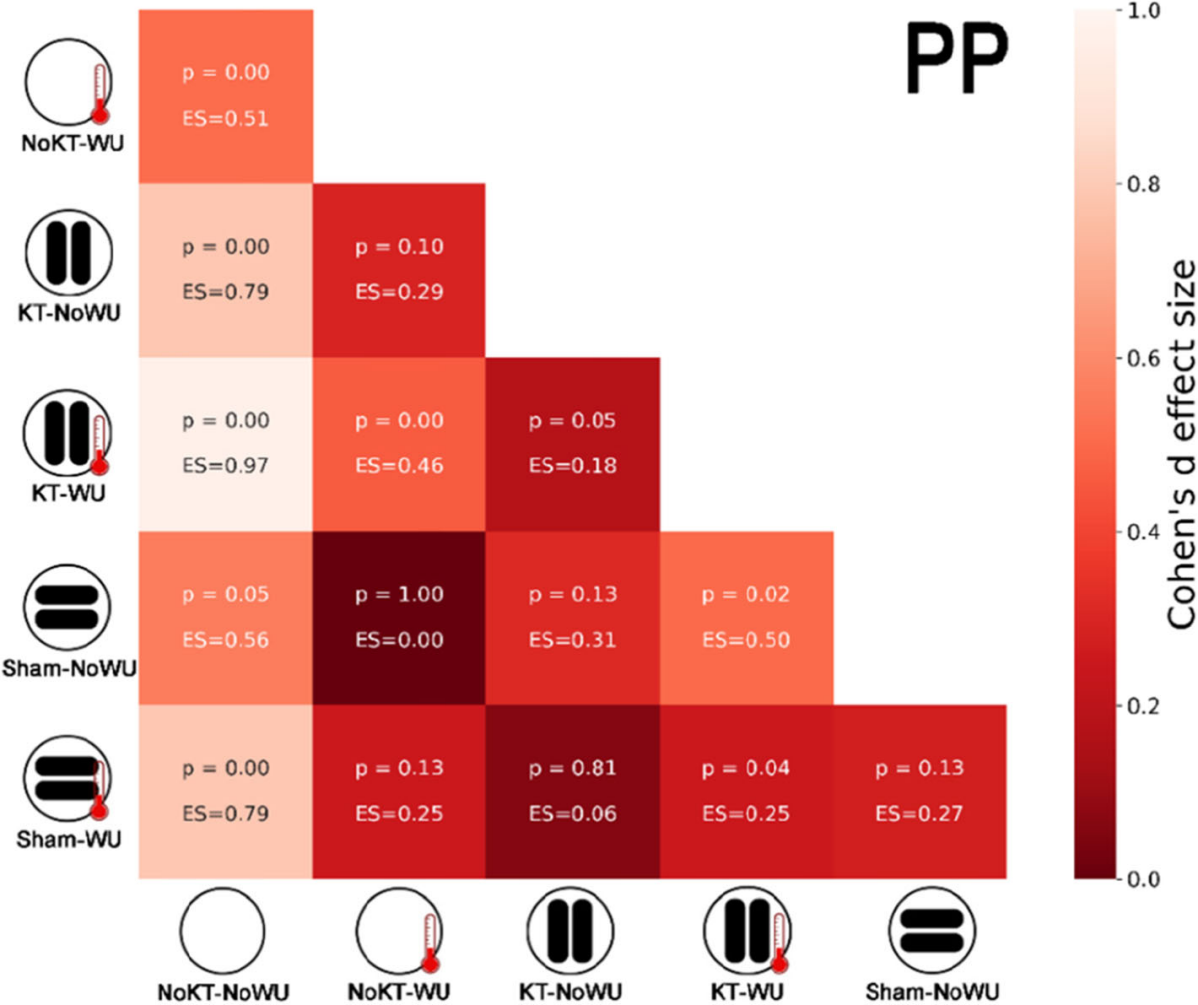
Heatmap showing Cohens’*d* effect sizes (ES) and *p*-values (p) of the pairwise comparisons between conditions in peak power (PP). Qualitative interpretation of the color scale is based on Cohens’*d* effect sizes. Note: KT = kinesio taping; WU = warm-up

**Fig. 4:**
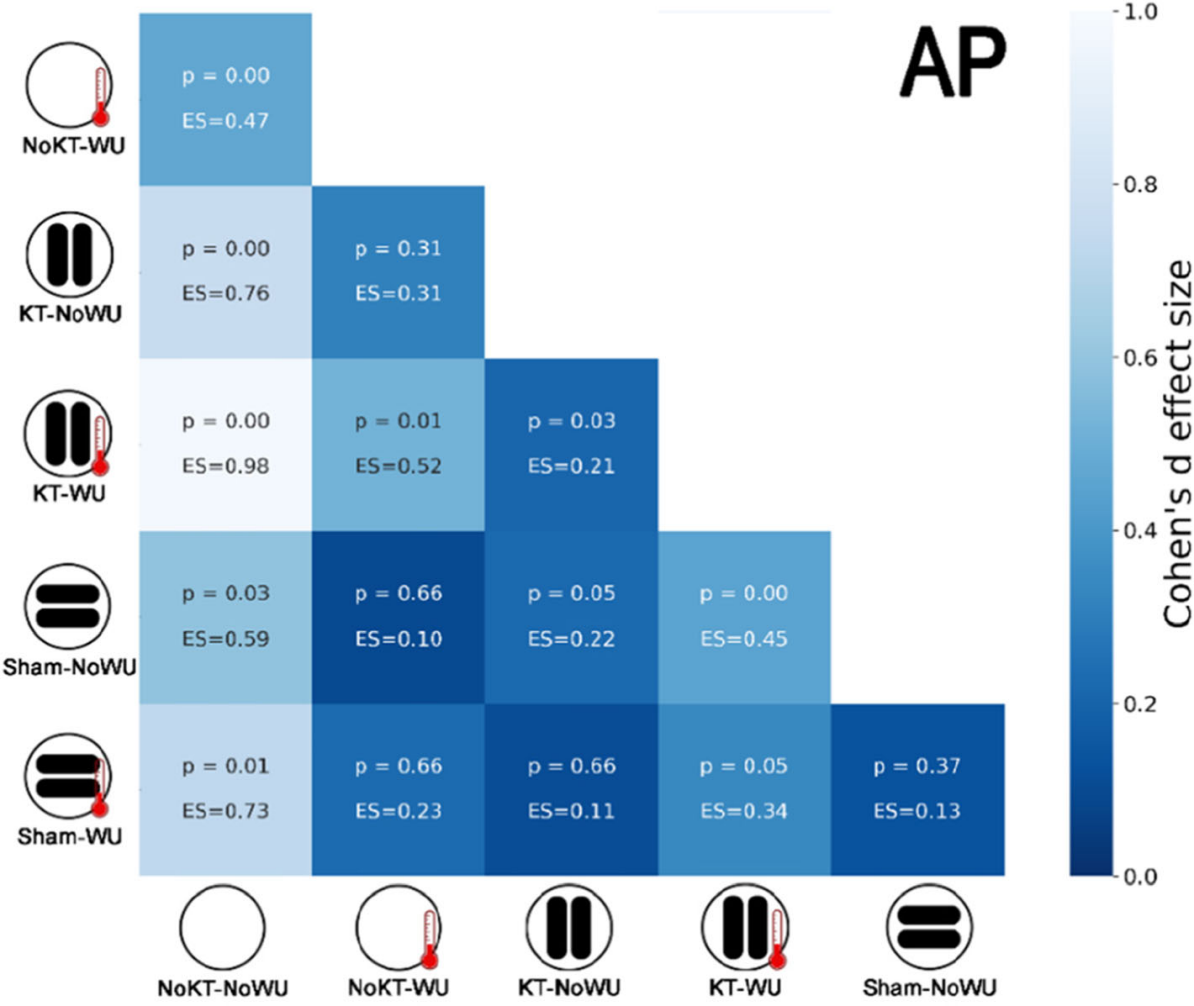
Heatmap showing Cohens’*d* effect sizes (ES) and *p*-values (p) of the pairwise comparisons between conditions in average power (AP). Qualitative interpretation of the color scale is based on Cohens’*d* effect sizes. Note: KT = kinesio taping; WU = warm-up

Figure 2 shows boxplots of PP and AP for each condition, together with pairwise comparisons. Significant differences between conditions (Fig. 2) were detected for both PP (F_(5,70)_ = 18.77, *p* < 0.001, observed power = 0.98) and AP (F_(5,70)_ = 15.91, *p* < 0.001, observed power = 0.99). In particular, KT-WU demonstrated the highest PP and AP with respect to the other conditions, except for AP that was similar to Sham-WU. Furthermore, NoKT-NoWU showed the lowest PP and AP values with respect to the other conditions. Heatmaps in the Figs. 3 and 4 show the Cohen’s *d*-values for PP and AP, respectively. Interestingly, the higher PP and AP values of KT-WU (*p* < 0.05) than KT-NoWU exhibited *small* effect sizes. Similarly, *small* effect sizes were also found between Sham-NoWU and Sham-WU in both PP and AP, together with no significant differences between the two Sham conditions (*p* > 0.05). Regarding NoKT-WU, PP was higher than NoKT-NoWU (*p* < 0.05) with a *moderate* effect size, whereas AP was higher than NoKT-NoWU (*p* < 0.05) with a *small* effect size.

## Discussion

The present study investigated the potential single and combined effect of KT and WU on sprint cycling performance by manipulating combinations of different KT conditions (with and without KT) and pre-exercise routine (with and without WU). The main finding of this study was that KT application promoted performance enhancement on maximal sprint cycling irrespective of pre-exercise routine, thus supporting our experimental hypothesis. Indeed, KT coupled with an active WU induced higher PP in sprint cycling with respect to the Sham and the No-KT conditions. This also occurred for AP, with the exception of Sham-WU that resulted in similar values to KT-WU. Overall, these findings provide support for the notion that KT might be a possible tool to be combined with the active WU routine, inducing benefit on sprint performance. Moreover, our results appear to suggest KT as an additional strategy to include within a passive WU, perhaps where an active WU is not feasible.

To the extent of our knowledge, this study is the first providing preliminary evidence on the KT application potentiality coupled with pre-exercise routine on a short-term maximal performance. After an active WU, participants with KT in situ presented higher PP and AP than NoKT condition with *small*-to-*moderate* effects (ES = 0.46 and 0.52). It is worth noticing that the active WU employed before sprinting was identical for both KT and NoKT. By this means, it appears the application of KT promoted further beneficial effects during sprint cycling performance. Interestingly, the present finding is consistent with previous results showing the KT effectiveness to enhance maximal sprint cycling performance [11, 13, 21]. For instance, the works of Trecroci and colleagues [11, 13] sought to investigate acute and short-term delayed effects of KT application over vastus medialis and vastus lateralis on sprint cycling performance. Overall, the authors found KT superior to induce higher PP outputs than NoKT immediately after its application and ~ 30 min afterwards [11, 13]. According to Slupik et al. [22], KT application may induce a reflex effect on the nervous system, which presumably might lead to a change in the front tight muscle tone. This seems to apply also for other muscle groups. In fact, Lumbroso et al. [23] observed a significant immediate peak force increase in the gastrocnemius muscle after KT application, while Huang et al. [24] reported higher ground-based reaction force and bioelectrical activity after KT application on the muscle group in healthy young adults. All together, these results would contribute to explain the higher PP values observed after KT-WU with respect to the other conditions.

A related idea which might explain this outcome is attributed to the supposed features of KT to induce tactile stimulation. It has been theorized that such tactile stimulation may be capable of activating cutaneous mechanoreceptors, thus, facilitating motor firing unit [25]. Although still debated, this mechanism is posited to improve muscle function [22, 26]. This may be supported by the finding of no difference (*p* > 0.05) between Sham and NoKT combined with WU (*small* ES). Additionally, whilst AP of KT-WU and Sham-WU presented similar values (*small* ES), their difference was barely non-significant (*p* = 0.05) perhaps masking a certain KT superiority. However, caution should be applied when interpreting the present findings. Indeed, although outside of the scope, this study did not include bioelectrical measures of the muscle functions at the site of tape application. This setting would provide, at least partially, further evidence to strengthen or weaken our extrapolations. Moreover, on top of this, the contradictory findings of KT effects on lower limb muscle strength [27, 28] suggest the need for supplementary research in order to clarify the rationale for KT usage in both pre-exercise and exercise routines.

Regarding the absence of WU, the ESs tended to get bigger (ES ≥ 0.79) when comparing KT with NoKT conditions. From this result, it may be inferred the capability of KT to influence participants’ sprint performance by acting as sort of passive WU. Of note, such an interpretation should be applied with caution as no physiological measures (core and skin temperatures) were employed. In this wake, it has been previously proposed the theoretical frame [12] based on the attributed effect of KT to improve blood microcirculation by the formation of convolutions along the stretched skin underlying the tape. These convolutions are supposed to increase the interstitial space between skin and connective tissues providing a better capillary perfusion. The improvement of perfusion, which is associated to skin temperature changes [8, 29], might have contributed to apply heat on the body tissues. However, it is worth noticing that results from Sham-NoWU and NoKT-NoWU seemed to yield no proof of this claim. Indeed, Sham exhibited higher PP and AP than NoKT with *moderate* ESs. Of note, as seen for KT, ESs tended to increase when comparing Sham with NoKT without WU.

On one hand, this indicates that KT and Sham behaved similarly against NoKT, which may be upheld by the existing *small* difference (*p* > 0.05) between KT-NoWU and Sham-NoWU. On the other hand, Sham application is not supposed to be responsible of the formation of convolutions along the underlying skin [11, 16]. Thereby, other reasons, rather than the mentioned theory, might be sought to explain this finding. For instance, it would be less surprising if we consider the presence of a psychological mediation [30]. Accordingly, an increased perception of strength was observed in healthy volunteers irrespective of the tape condition, with ~ 45 and 30 % of them feeling stronger after KT and placebo applications, respectively [31]. Of note, the physical difference between how the strips appears (e.g., the direction in which the tape or strips are positioned, and the amount of tension applied to them) may affect the perception of wellbeing, safety and strength, perhaps controlling for potential placebo effects [31, 32]. Taken altogether, these considerations may help to feature a plausible interpretation of the current results.

The present study has some limitations that should be clarified. Neither double-blinded procedure (i.e., athletes and experimenters not conscious about the conditions) nor physiological analysis (i.e., core, skin and muscle temperature and bioelectrical muscle signals) have been employed within the study. The lack of double-blinding procedure could result in a potential psychological effect that might impact sprint performance. Moreover, the absence of physiological data does not permit a deep understanding of the underlying mechanisms related to WU and KT effects on sprint performance. Therefore, our findings should not be over-interpreted and generalized. Future research will have to deeply look at the actual physiological mechanisms of actions underlying the KT application combined with WU routine also to ascertain the veracity of its attributed effects in relation to sport performance.

## Conclusions

In conclusion, the main finding of this study was that KT application induced enhancement on maximal sprint cycling performance regardless of the presence of an active WU. Although the role of KT in influencing muscle function, skin temperature (by an increased capillary perfusion) and psychological attributes should be further elucidated, this study highlights the potentiality of KT for enhancing sprint performance. From practical perspectives, these findings demonstrated that KT might be a possible tool to be coupled with an active WU routine. Moreover, whether active WU is not feasible, KT might be considered a potential strategy to include within a passive WU.

## Data Availability

All relevant data were provided throughout the paper.

## Abbreviations

AP: Average power
ES: Effect size
ICC: Intraclass correlation coefficient
KT: Kinesio taping
NoKT: Without kinesio taping
NoWU: without warm-up
PP: Peak power
SD: Standard deviation
WU: Warm-up
